# Salivary 8-hydroxyguanine as a lifestyle-related oxidative stress biomarker in workers

**DOI:** 10.3164/jcbn.19-72

**Published:** 2020-01-01

**Authors:** Sintaroo Watanabe, Yuya Kawasaki, Kazuaki Kawai

**Affiliations:** 1 Department of Environmental Oncology, Institute of Industrial Ecological Sciences, University of Occupational and Environmental Health, Japan, 1-1 Iseigaoka, Yahatanishi-ku, Kitakyushu-shi, Fukuoka 807-8555, Japan; 2 Japan Marine United Corporation Kure Shipyard, 2-1 Showa-cho, Kure-shi, Hiroshima 737-0027, Japan

**Keywords:** 8-hydroxyguanine, saliva, oxidative stress, biomarker, lifestyle

## Abstract

Oxidative stress is a risk factor for lifestyle-related diseases, such as cancer. Investigations of the factors that increase or decrease oxidative stress contribute to disease prevention. In the present study, we focused on the 8-hydroxyguanine (8-OHGua) in saliva, as a new oxidative stress biomarker. The relationship between lifestyles and salivary 8-OHGua levels in 541 Japanese subjects was analyzed. The salivary 8-OHGua levels were significantly elevated in older persons, as well as those who smoke, have hypertension, or excess visceral fat. By contrast, statistically significant lower levels of 8-OHGua were observed in persons who moderately exercised or recently drank green tea or coffee. The direct collection of saliva, without any special collecting device, was suitable for the 8-OHGua analysis. The present results suggest that oxidative stress can be measured in a non-invasive manner with easily collectable saliva, and the salivary 8-OHGua may be a useful biomarker for lifestyle-related disease prevention.

## Introduction

**Fig. 1:**
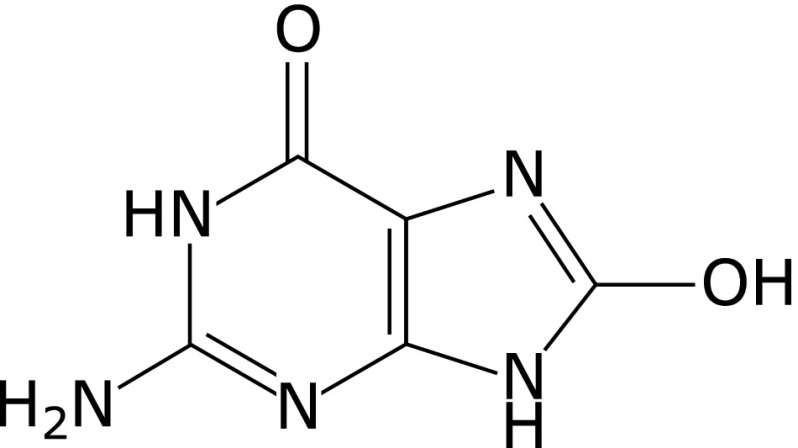
The structural formula of 8-OHGua.

Oxidative stress is involved in lifestyle-related diseases, such as cancer.^(1)^ One of the most representative oxidative stress markers is 8-hydroxy-2'-deoxyguanosine (8-OHdG), which reflects the oxidative damage of the nucleobase, and has been widely analyzed using non-invasively collected urine samples. It is well known that urinary 8-OHdG values are increased by aging, smoking, alcohol consumption, and other factors.^(2)^ The free base, 8-hydroxyguanine (8-OHGua, Fig. 1), is also detectable in biological fluids, such as urine^(3–6)^ and serum.^(4)^ The 8-OHGua is produced by the base excision repair of oxidized DNA or by the oxidation of a free guanine base.^(7)^ The application of 8-OHGua as an oxidative stress marker is presently still limited. We recently established an analytical method for salivary 8-OHGua, using HPLC equipped with an electro chemical detector (ECD).^(8)^ The 8-OHdG level in saliva is difficult to measure accurately, due to its low concentration.^(8)^ Several salivary biomarkers are reportedly useful in the diagnoses of systemic diseases, such as cardiovascular lesions, cancer, and diabetes,^(9,10)^ but few studies have investigated the relationship between lifestyle and salivary antioxidant capacity.^(11)^ The aim of this study was to evaluate the relationship between oxidative stress and lifestyle in workers, by measuring the salivary 8-OHGua levels. Saliva has been drawing attention as a biological monitoring matrix, especially because of its easy and noninvasive collection. However, the measurement results are crucially dependent on the sampling method.^(12)^ One high priority issue to be solved is the potential interaction of the material in the collection device with the target analyte.^(13)^ Therefore, the material composition of the saliva collection device was also evaluated in this study.

## Materials and Methods

### Saliva Collection

#### Experiment 1: Evaluation of the Saliva Sampling Method

 Ten min after rinsing the mouth with water, 2 ml of saliva were collected in three different ways, as follows: direct collection into a polypropylene tube [passive drool (PD)], commercially available material [SalivaBio Oral Swab (SOS), Salimetrics, L.L.C., Carlsbad, CA], and surgical cotton (Hakujuji Co., Ltd., Tokyo, Japan). In order to detect the absorption of 8-OHGua to the collection materials, samples of about 5 ml of PD saliva were collected from 7 persons. The collected saliva was divided into 3 portions. The saliva-soaked SOS or cotton samples were left to adsorb for 10 min at room temperature. The absorbed saliva was recovered from the soaked SOS or cotton by centrifugation at 3,500 rpm for 10 min.

#### Experiment 2: Worker Lifestyle and Oxidative Stress

A total of 635 volunteers (602 male and 33 female) aged 18–64, from three companies in Japan, participated in the study. After excluding samples because of sputum or blood contamination in the saliva, 541 subjects (513 male and 28 female) were selected for analysis. After rinsing the mouth with water, about 2 ml of PD saliva was collected into a polypropylene tube in the morning (8 A.M.–11 A.M.). The collected saliva samples were kept in an ice cooler box during the sample collection, and then frozen at −30°C until analysis. At the same time, the following information was obtained by a questionnaire. The survey covered items such as gender, age, smoking status, alcohol consumption, green tea or coffee intake, daily physical activity, hypertension, waist circumference, and body weight gain compared with weight at age at 20.

### Analysis of 8-OHGua

Each saliva sample was stored at −30°C until analysis. Fifteen µl (Experiment 1) or 5 µl (Experiment 2) of Proteinase K (20 mg/ml in water, Wako Chemical, Tokyo, Japan) were added to 300 µl (Experiment 1) or 100 µl (Experiment 2) of saliva. The mixture was incubated at 37°C for 1 h. The mixture was evaporated to dryness by centrifugal vacuum concentration, and the residue was dissolved in 300 µl of dilution solution (1.8% acetonitrile, 62 mM NaOAc, 0.01 mM H_2_SO_4_). After filtration through a centrifugal filter (Amicon Ultra, Ultracal-10K, Merck Millipore Ltd., Darmstadt, Germany), a 20 µl portion of the filtrate was analyzed by an HPLC system to determine the 8-OHGua concentration. Measurements of 8-OHGua levels were performed based on the method of Kawai *et al.*^(8)^

The study was approved by the University of Occupational and Environmental Health Ethics Committee. Written informed consent was obtained from all subjects.

### Statistical Analyses

All statistical analyses were performed with the EZR statistical software (Saitama Medical Center, Jichi Medical University, Saitama, Japan),^(14)^ which is a graphical user interface for R (The R Foundation for Statistical Computing, Vienna, Austria). The Wilcoxon signed-rank test was used for experiment 1. The statistical methods for experiment 2 are shown in each figure. The statistical significance level was set at 5% (*p*<0.05).

## Results

### Experiment 1

**Fig. 2:**
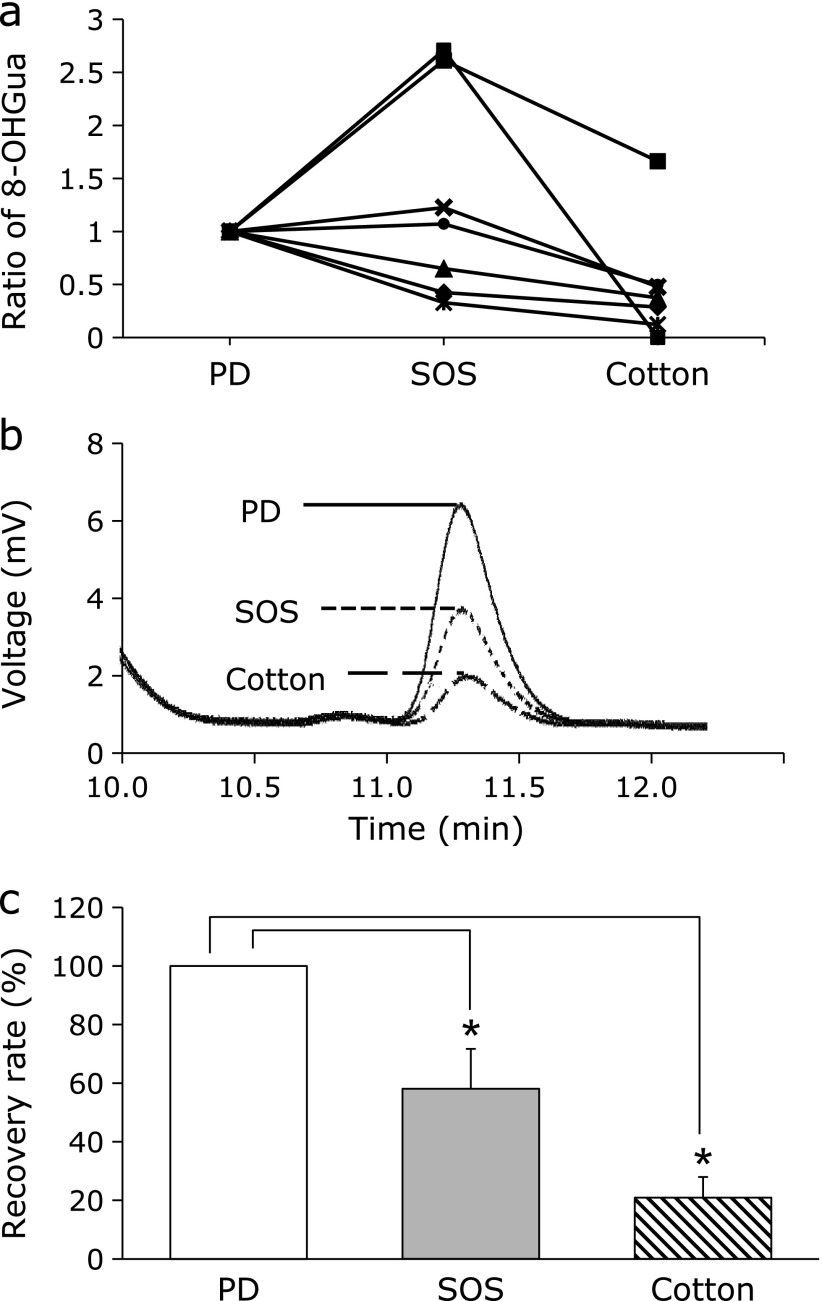
8-OHGua levels in saliva analyzed by HPLC-ECD. (a) The ratio of 8-OHGua measurement results (PD to collection material, SOS or cotton) for each subject (*n* = 7). (b) Typical HPLC-ECD chromatograms of the 8-OHGua in PD, SOS, and cotton samples. (c) Recovery rates of 8-OHGua in the PD, SOS, and cotton groups. Columns represent mean ± SE (*n* = 7). *p<0.05 (Wilcoxon signed-rank test).

The salivary 8-OHGua measurement results were affected by the usage of the saliva absorption materials, such as SOS or surgical cotton (Fig. 2a). In the case of SOS, the absorption levels of 8-OHGua varied widely (from 32 to 271% of PD) among the different subjects. The use of surgical cotton led to lower 8-OHGua values in most subjects. To investigate the underlying causes of these effects, the 8-OHGua levels of saliva soaked into SOS or cotton were measured. A typical HPLC-ECD chromatogram of 8-OHGua in saliva soaked into SOS or cotton is shown in Fig. 2b. The recovery rates of 8-OHGua from SOS or cotton were 58.1% and 20.9% of PD (Fig. 2c).

### Experiment 2

**Fig. 3:**
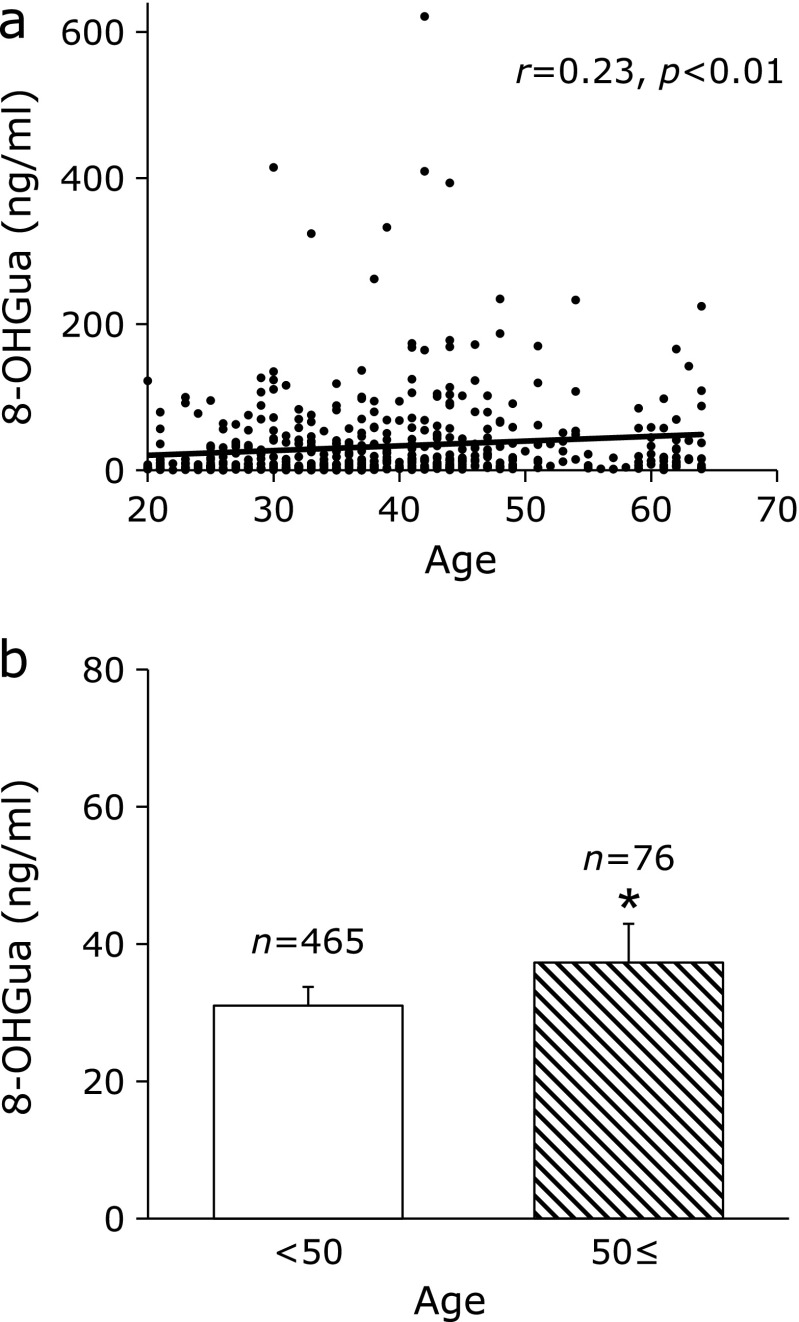
Age and salivary 8-OHGua level. (a) Association between age and salivary 8-OHGua level. *r* represents the Spearman’s rank correlation coefficient. (b) Salivary 8-OHGua levels in subjects under 50 and over 50 years old. Each column represents mean ± SE. *p<0.05 (Mann–Whitney test).

**Fig. 4:**
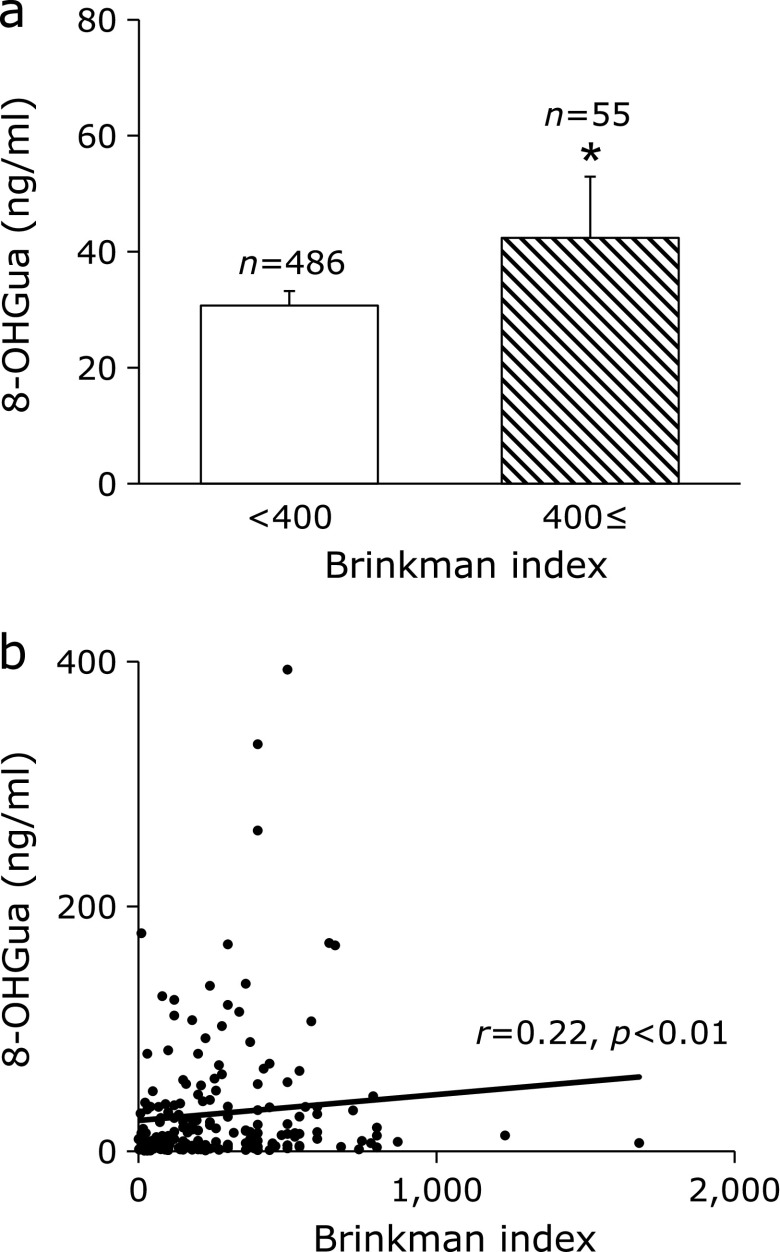
Smoking and salivary 8-OHGua level. (a) Salivary 8-OHGua levels in subjects with Brinkman index ≥400 and Brinkman index <400. Columns represent mean ± SE. *p<0.05 (Mann–Whitney test). (b) Association between current smokers’ Brinkman index and the salivary 8-OHGua level. *r* represents Spearman’s rank correlation coefficient.

**Fig. 5:**
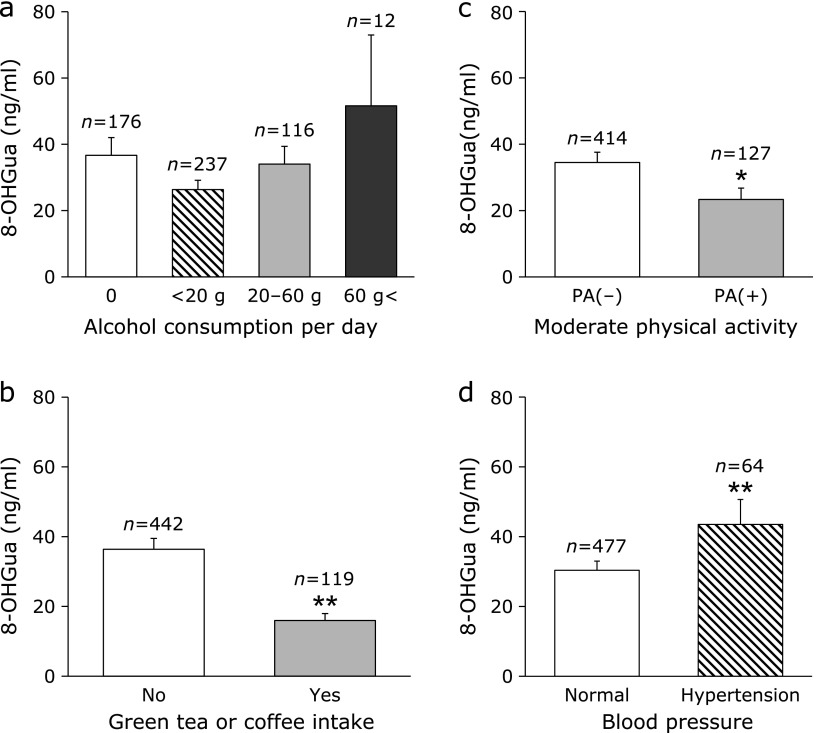
Lifestyle-related factors and salivary 8-OHGua levels. (a) Alcohol consumption and salivary 8-OHGua. (b) Salivary 8-OHGua level and green tea or coffee consumption (within 48 h before saliva collection). (c) Salivary 8-OHGua level and daily physical activity. “PA (–)” represents subjects that don’t perform physical activity, and “PA (+)” represents subjects that perform physical activity, like walking, on a daily basis. (d) Salivary 8-OHGua level and hypertension. Each column represents mean ± SE. *p<0.05, **p<0.01 [Mann–Whitney test for (b), (c), and (d)].

**Fig. 6:**
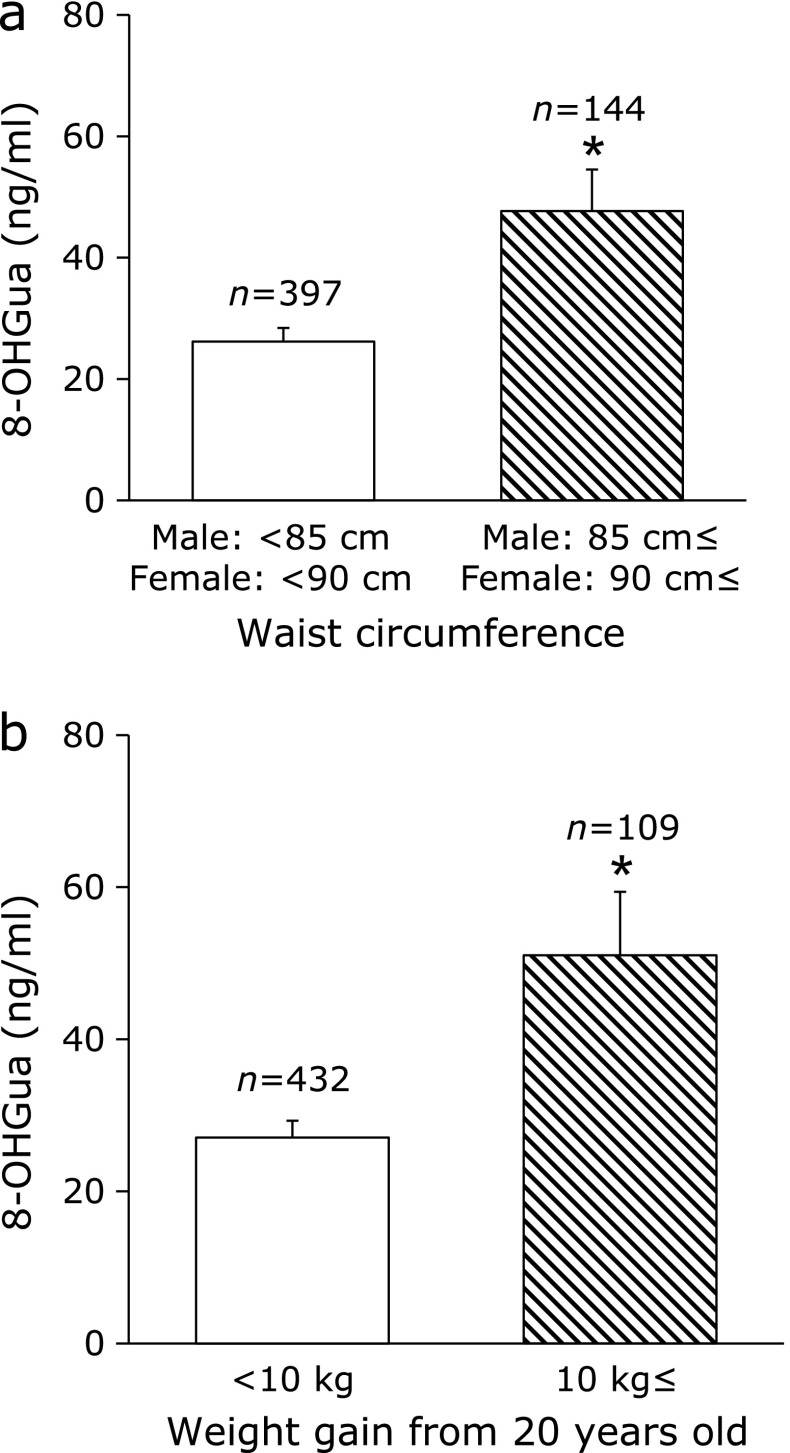
Obesity and salivary 8-OHGua level. (a) Waist circumference and salivary 8-OHGua level. (b) Weight gain and salivary 8-OHGua. Each column represents mean ± SE. *p<0.01 (Mann–Whitney test).

As the accuracy of the measurement, the coefficient of variation calculated based on the 8-OHGua standard solution was within 5%. The mean 8-OHGua level (ng/ml saliva) in 541 subjects was 31.9 (minimum: 0.10, maximum: 621.0, median: 10.4). There was no significant difference between male and female. A weak positive correlation was found between age and salivary 8-OHGua level (Fig. 3a). When separated at 50 years of age, the salivary 8-OHGua level was significantly higher in the group of persons aged 50 years and older, as compared to that in the under 50 group (Fig. 3b). In terms of smoking, the salivary 8-OHGua level was significantly higher in the group of subjects with a Brinkman index (BI: number of cigarettes smoked per day × number of years of smoking) ≥400, as compared to that in the group with BI <400 (Fig. 4a). In addition, in the case of current smokers, a weak positive correlation between the BI value and 8-OHGua was found (Fig. 4b). Even though there was no statistically significant difference, the 8-OHGua level increased with the daily consumption of alcohol (Fig. 5a). Interestingly, the 8-OHGua level of non-drinkers was higher than that of light drinkers. Subjects who consumed green tea or coffee within 48 h before the saliva collection had significantly lower 8-OHGua levels, as compared with subjects who did not consume them (Fig. 5b). The 8-OHGua level was significantly lower in the group of persons who were physically active on a daily basis, as compared with the subjects who did not do physical activity (Fig. 5c). Hypertensive subjects had significantly higher 8-OHGua levels, as compared to those without hypertension (Fig. 5d). Visceral fat obese subjects (waist circumference: 85 cm≤ for male, 90 cm≤ for female; the Japanese diagnostic criteria for metabolic syndrome) had significantly higher levels of 8-OHGua, as compared with non-visceral fat obese subjects (Fig. 6a). In addition, the 8-OHGua level was significantly higher in subjects who gained more than 10 kg of body weight since they were 20 years old (a risk factor of metabolic syndrome) than those who maintained their weight (Fig. 6b).

## Discussion

When saliva is used as a biomonitoring matrix, the material in the collecting device is one of the major factors influencing the levels of biomarkers. For example, the levels of thiobarbituric acid reacting substances,^(15)^ testosterone, DHEA (Dehydroepiandrosterone), progesterone, and estradiol^(16)^ were artifactually increased by the use of a cotton-based collection material. In contrast, glutathione^(17)^ and sIgA (secretory Immunogloblin A)^(16)^ levels were reduced by cotton. The use of a polypropylene-based material lowered the cortisol levels in samples.^(15)^ In saliva samples collected with synthetic material, the levels of amylase, IgA, and lactoferrin were low.^(18)^ For the accurate measurement of 8-OHGua, the appropriate collection methods were evaluated at the beginning of this study. As shown in Fig. 2c, 8-OHGua was absorbed to SOS or cotton in the PD saliva samples collected from the same subjects in Fig. 2a. The absorption of 8-OHGua to SOS or cotton is considered as a major cause of the low 8-OHGua levels in Fig. 2a. These results suggested that the most appropriate method is PD for the measurement of 8-OHGua. Futher analysis is needed to determine the reason for the higher value of 8-OHGua in the SOS-collected saliva. The flow rate of the saliva might have been disturbed by the collection materials. As a result, the secretion of 8-OHGua into the saliva might be affected.

Reactive oxygen species (ROS) are implicated as a cause of cancer and lifestyle-related diseases.^(2,19)^ The practical effectiveness of salivary 8-OHGua as a new oxidative stress marker was examined in workers, in experiment 2. ROS seem to be one of the key factors in aging.^(20)^ The urinary 8-OHGua and 8-OHdG levels increased with age in mice.^(21)^ In humans, oxidative damage biomarkers of DNA (8-OHdG), lipids (malondialdehyde), and proteins (carbonyl compounds) in skeletal muscle increased age-dependently.^(22)^ The 8-OHdG levels in leukocyte DNA and urine were significantly higher in elderly subjects.^(23)^ In this study, a relatively weak positive relationship was observed between age and salivary 8-OHGua in the scatter plots. In the cases separated by age (50 years old), the salivary 8-OHGua levels were significantly higher in the group of subjects aged 50 or over. The salivary 8-OHGua levels may be associated with aging. These results suggested that the salivary 8-OHGua level may be a useful biomarker of aging. Several molecular epidemiologic studies revealed that oxidative damage plays a certain role in inducing human lung cancer by smoking. The 8-OHdG levels in sperm^(24)^ were higher in smokers. The Brinkman index and the 8-OHdG level in lung tissue DNA showed a positive correlation.^(25)^ The urinary 8-OHdG levels were also higher in smokers.^(26)^ Our finding of the positive relation between the salivary 8-OHGua levels and the Brinkman index agreed well with previous studies. Regarding the alcohol-related diseases, several cohort studies have shown a dose-dependent increase in cirrhosis risk with high alcohol consumption (40 g/day for women and 60 g/day for men).^(27)^ On the other hand, the mortality of moderate drinkers (less than 23 g/day) was lower than that of nondrinkers.^(28)^ Several reports have shown a J-shaped relationship between alcohol consumption and related diseases.^(29,30)^ Interestingly, the variations of the salivary 8-OHGua levels in this study were quite similar to those in previous reports. Alcohol-induced oxidative stress is a major cause of related diseases.^(31)^ Many kinds of antioxidants, such as an astaxanthin,^(32)^ lycopene,^(33)^ green tea polyphenols,^(34)^ green tea aroma,^(35)^ and coffee,^(36)^ were reported to reduce oxidative stress. In the present study, the salivary 8-OHGua levels were decreased by green tea or coffee consumption. Moderate physical activity also reduced oxidative stress.^(37,38)^ At the same time, GSH (Glutathione-SH), catalase, glutathione peroxidase activity,^(2,39)^ and total antioxidant capacity^(38)^ were increased. The salivary 8-OHGua levels were significantly lower in persons who performed moderate physical activity. Metabolic syndrome, which involves several metabolic risk factors such as obesity, insulin resistance, hypertension, and dyslipidemia, has become a serious problem in developed countries. Many reports support the idea that increased oxidative stress plays an important role in metabolic syndrome-related diseases.^(40)^ The present findings of high salivary 8-OHGua in hypertensives are consistent with previous studies that suggested a link between oxidative stress and hypertension.^(2,41)^ Therefore, the salivary 8-OHGua level may reflect the oxidative stress due to hypertension. Based on data from 2013, it was estimated that 4.5 million deaths worldwide were caused by excessive weight and obesity. The IARC (International Agency for Research on Cancer) working group concluded that the absence of excess body fat lowers the risk of most cancers.^(42)^ In this study, the salivary 8-OHGua levels were significantly higher in the subjects above the standard Japanese diagnostic criteria (waist circumference and weight gain) for metabolic syndrome. The salivary 8-OHGua may be a suitable index reflecting the oxidative stress due to obesity.

To the best of our knowledge, this is the first study to investigate the relationship between workers’ lifestyles and the levels of salivary 8-OHGua as an oxidative stress biomarker. Among the several biological fluids potentially available for oxidative stress measurement, saliva has been attracting interest for a long time, because of its non-invasive collection. The avoidance of mental and physical pain during sampling provides benefits to the subjects, medical service workers, and researchers. The best advantage is that saliva contains an adequate amount of 8-OHGua for the accurate measurement. These reasons highlight the benefits of using the combination of saliva as a sample and 8-OHGua as an oxidative stress marker. On the basis of our results, the salivary 8-OHGua is a useful biomarker to evaluate the oxidative stress for the prevention of lifestyle-related diseases.

## Author Contributions

SW, YK, and KK collected the samples and data. SW statistically analyzed the data. KK and SW designed and critically discussed the study.

## Abbreviations

8-OHGua: 8-hydroxyguanine
8-OHdG: 8-hydroxy-2'-deoxyguanosine
PD: passive drool (directly collected saliva)
SOS: SalivaBio Oral Swab
